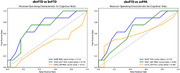# ASSESSING DYNAMIC AFFECT RECOGNITION FOR DISTINGUISHING FRONTOTEMPORAL DEMENTIA SUBTYPES

**DOI:** 10.1002/alz.091990

**Published:** 2025-01-03

**Authors:** Hulya Ulugut

**Affiliations:** ^1^ Memory & Aging Center, Department of Neurology, University of California in San Francisco, San Francisco, CA USA

## Abstract

**Background:**

Recent international work suggests that more precise subtyping within frontotemporal dementia (FTD) syndromes leads to better prediction of pathology, supporting individualized disease‐specific treatments. Recent studies emphasize that identification of one such subtype, semantic behavioral variant FTD (sbvFTD), relies in part on measuring emotion recognition abilities.

**Method:**

In order to evaluate the effectiveness of current tools, we compared the brief video‐based Dynamic Affect Recognition Test (DART) against the TASIT Emotion Evaluation (EET) and Comprehensive Affect Testing System Affect Matching tests. 105 individuals in the very early stages (CDR≤1) of FTD (61 behavioral variant FTD [bvFTD], 25 semantic variant primary progressive aphasia [svPPA], 19 sbvFTD), and 71 healthy older participants underwent emotion testing and structural MRI.

**Result:**

Tests using dynamic (video) stimuli outperformed static face stimuli for distinguishing sbvFTD from bvFTD, but the DART outperformed the EET distinguishing sbvFTD from svPPA (Figure 1). Structural VBM analysis of DART performance showed predominant contribution of right‐sided structures involved in semantics and attention (e.g. temporal pole, insula), though in sbvFTD, who had worse DART scores than all other syndromes (p<0.05), DART performance also corresponded with temporo‐orbitofrontal structures mediating hedonic evaluation.

**Conclusion:**

Our findings indicate that emotion reading tests using dynamic stimuli are best for differentiating among FTD syndromes. Also, unique profiles of emotion reading deficits emerging from different syndromes correspond to distinct patterns of damage to emotion, semantics, attention, and reward‐related brain networks. Emotion identification deficits are a core feature of FTD, therefore careful selection of tests that reflect the key underlying neural circuits will result in more accurate phenotyping.